# Fluoroscopically guided vascular and cardiac transcatheter procedures: a comparison of occupational and patient dose by anatomical region

**DOI:** 10.1007/s13246-023-01226-7

**Published:** 2023-03-06

**Authors:** Kelly S. Wilson-Stewart, Davide Fontanarosa, Eva Malacova, Jamie V. Trapp

**Affiliations:** 1 School of Chemistry and Physics, Faculty of Science, Queensland University of Technology, 2 George Street, Brisbane, QLD 4000 Australia; 2 Cardiovascular Suites, Greenslopes Private Hospital, Ramsay Health Care, Newdegate Street, Greenslopes, Brisbane, QLD 4120 Australia; 3 School of Clinical Sciences, Faculty of Health, Queensland University of Technology, 2 George Street, Brisbane, QLD 4000 Australia; 4 Centre for Biomedical Technologies (CBT), Queensland University of Technology, 149 Victoria Park Road, Kelvin Grove, Brisbane, QLD 4059 Australia; 5 QIMR Berghofer Medical Research Institute, 300 Herston Road, Herston, QLD 4006 Australia

**Keywords:** Occupational exposure, Scrub nurse, Circulator nurse, Patient dose, Fluoroscopic imaging, X-ray imaging

## Abstract

X-ray guided procedures are being performed by an increasing variety of medical specialties. Due to improvements in vascular transcatheter therapies, there is an increasing overlap of imaged anatomy between medical specialties. There is concern that non-radiology fluoroscopic operators may not have sufficient training to be well informed of the potential implications of radiation exposure and mitigation strategies to reduce dose. This was a prospective, observational, single center study to compare occupational and patient dose levels when imaging different anatomical regions during fluoroscopically guided cardiac and endovascular procedures. Occupational radiation dose was measured at the level of the temple of 24 cardiologists and 3 vascular surgeons (n = 1369), 32 scrub nurses (n = 1307) and 35 circulating nurses (n = 885). The patient dose was recorded for procedures (n = 1792) performed in three angiography suites. Abdominal imaging during endovascular aneurysm repair (EVAR) procedures was associated with a comparatively high average patient, operator and scrub nurse dose despite additional table-mounted lead shields. Air kerma was relatively high for procedures performed in the chest, and chest + pelvis. Higher dose area product and staff eye dose were recorded during procedures of the chest + pelvis due to the use of digital subtraction angiography to evaluate access route prior to/during transaortic valve implantation. Scrub nurses were exposed to higher average radiation levels than the operator during some procedures. Staff should be cognizant of the potentially higher radiation burden to patients and exposed personnel during EVAR procedures and cardiac procedures using digital subtraction angiography.

## Introduction

Historically, x-ray guided procedures were performed within imaging departments by radiologists, but recent advances in treatment options and equipment have seen fluoroscopic imaging being utilized within an ever-increasing range of medical specialities [1, 2]. The treating specialty depends on the type and location of the patient’s pathology. Interventional neuro-radiologists perform procedures on the head or the spine. Interventional radiologists typically conduct procedures on abdominal organs and vasculature, gastroenterologists image the abdomen (abdo), vascular surgeons may visualize vessels in the arms, legs, and torso, and cardiologists typically image the heart. There is an overlap between the imaged anatomy in several different medical specialties. For example, it is routine for vascular surgeons to image the chest as part of a thoracic endovascular aneurysm repair (TEVAR). Conversely, cardiologists may perform renal angiography as part of an investigation into unexplained hypertension [3], or image pelvic arteries as part of a transaortic valve implantation (TAVI) [4].

As the numbers and types of procedures performed by non-radiologists grow, so does the need for dedicated fluoroscopic suites beyond their traditional location within the medical imaging department and, as a consequence, there may be less awareness of the potential radiation risks and mitigation strategies by the staff performing them [2, 5]. Due to the potentially detrimental effects to both the patient and staff involved [6], there are justifiable concerns over the radiation exposure during fluoroscopic procedures.

Exposure to ionizing radiation may result in oncogenesis (considered a stochastic effect), but demonstrating a definitive link between an incidence of medical or occupational exposure and the formation of cancer is difficult due to the long latency period between the procedure and manifestation, and also the naturally high prevalence of cancer in the population [5]. Another potential effect is damage to tissues (deterministic effect). Deterministic effects typically have a threshold below which they will not occur [7]. Skin damage to patients following fluoroscopically guided procedures has been well reported [8, 9], as has the incidence of subcapsular cataracts in occupationally exposed staff [10, 11]. Unlike visible tissue changes to the skin or the eyes, it is more difficult to observe tissue changes in internal organs and vascular systems [6]. It has been reported that there may be a causal relationship between occupational exposure and circulatory diseases [12, 13], DNA damage [14], cognitive impairment [15, 16] and the formation of cancer [17]. There has been limited literature to date which investigates the differences in operator and nursing dose during fluoroscopic procedures undertaken by vascular surgeons and cardiologists. In addition, there is also scarce clinical studies comparing the effect of irradiated anatomical area on occupational and patient dose.

X-ray images are created by the detection of photons that are transmitted through a patient, as opposed to those attenuated by the tissues along the path of the primary beam [18]. The elemental composition of tissues affects the degree of absorption of the beam, with adipose tissue (which has a high content of hydrocarbon) being less likely to affect the trajectory of an x-ray photon than cortical bone, which contains calcium and phosphorus [19, 20]. With this in mind, theoretically, cardiologists and their patients should be exposed to lower levels of radiation than their endovascular counterparts due to the less dense tissues being irradiated within the chest, compared with the abdo and pelvis. This study aims to quantify and compare the levels of patient and staff dose for imaging of different anatomical regions during endovascular and cardiac transcatheter fluoroscopic procedures.

## Methods

This research compares staff eye dose and patient dose levels during fluoroscopic procedures performed within the chest, abdo, pelvis, arms and legs by cardiologists and vascular surgeons. Patient and occupational temple dose was prospectively recorded between February 2017 and August 2019 for cardiologists (n = 24), vascular surgeons (n = 3), scrub nurses (n = 32) and circulator nurses (n = 35) at a large tertiary hospital. Cardiology and vascular procedures were classified by imaged anatomical area (Table 1). Convenience sampling was used to source participants. All nursing staff were skilled in both cardiac and vascular procedures, and all operators had > 15 years’ experience in transcatheter procedures.

**Table 1:** A list of endovascular and cardiac trans-catheter procedures included in each of compared anatomical categories

Anatomical classification	Abbreviation	Examples of included procedures
Chest		Electrophysiology
		Closure device implantation
		Permanent pacemaker insertion/lead replacement
		Diagnostic/interventional coronary angiography
		Pericardiocentesis
		Subclavian diagnostic/interventional angiogram
Chest and Pelvis	Chest + Pelvis	TAVI
		TAVI workup
Abdo (non-EVAR)	Abdo	Calibrating aortogram
		Diagnostic/interventional mesenteric or renal angiography
		Aortogram and iliac intervention
		Balloon pump insertion
Abdo (EVAR)	EVAR	Endovascular aneurysm repair
Pelvis		Diagnostic/interventional pelvic aortogram
		Diagnostic/interventional Iliac angiography
Pelvis + single leg	Pelvis + SL	Pelvic aortogram with diagnostic/interventional single leg angiography
Pelvis + both legs	Pelvis + BL	Pelvic aortogram with diagnostic/interventional both leg angiography
Abdo + single leg	Abdo + SL	Abdominal aortogram with diagnostic/interventional single leg angiography
Abdo + both legs	Abdo + BL	Aortobifemoral diagnostic/interventional both leg angiography
		Iliac intervention with run-off both leg angiograms
Single leg-downhill	SL-DH	Antegrade ipsilateral approach single leg diagnostic/interventional angiography
Single leg-normal	SL-N	Retrograde contralateral approach single leg diagnostic/interventional angiography
Arm		Diagnostic/interventional dialysis fistulogram

*Abdo* abdomen; *TAVI* transcatheter valve implantation

The occupational eye dose was measured via the DoseAware dosimetry system (Philips Healthcare, Best, Netherlands). The DoseAware system consists of badges that are solid-state active personal dosimeters which log occupational radiation dose per second cumulatively [21]. DoseAware badges have been demonstrated to detect satisfactorily within varying pulse widths, tubevoltages and dose rates [22, 23] and have a reported uncertainty of 5% [24]. Manufacturer specifications state that DoseAware has a detectable dose range 1 μSv–10 Sv [24]*.* DoseAware provides appropriate estimates of eye dose when worn close to the eye and has been shown to respond satisfactorily in realistic scattered radiation fields [25, 26].

Procedures were performed using Philips Allura Xper angiographic equipment (Philips Healthcare, Best, Netherlands). All x-ray equipment underwent annual calibration and compliance testing by a medical physicist.

**Fig. 1:**
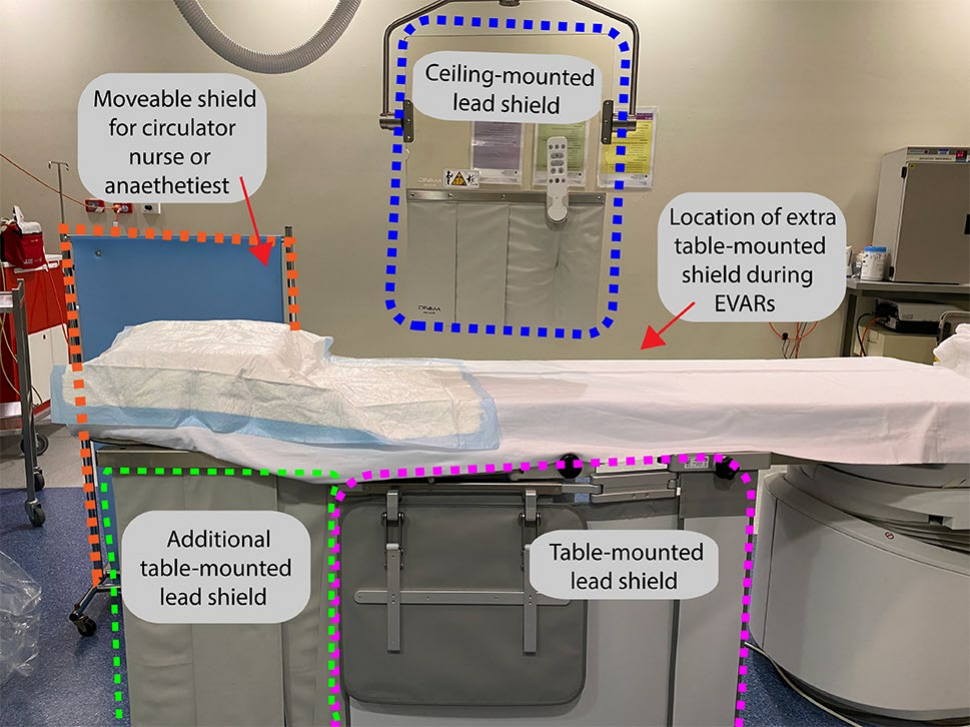
Typical room setup and shielding option including a mobile shield (blue) that can be altered depending on staff position in the room and an adjustable ceiling mounted lead shield to be positioned between the operator and the x-ray detector. *The table mounted shield used on the opposite side of the table for EVAR are the same style as those visualized in the figure

**Fig. 2:**
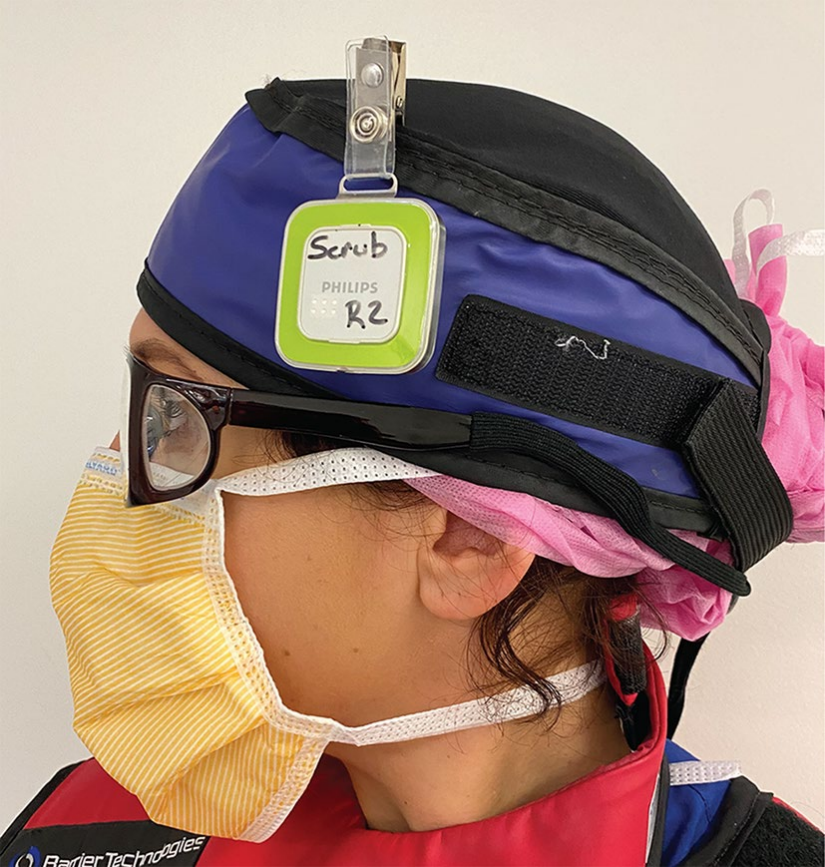
Location of DoseAware badge when attached external to skull cap. The dosimeter badge was worn on the temple closest to the x-ray tube gantry

Conventional room setup is demonstrated in Fig. 1. At least one lead shield was mounted on the operating side during all cases, with additional table-side mounted shielding, similar to the ones demonstrated in Fig. 1, utilized in cases that were anticipated to be higher dose or those with anaesthetists. Standard personal protective equipment consisted of a lead wraparound skirt, top and thyroid shield. It was typical for scrubbed staff to wear lead shin protectors and glasses, and the scrub nurses often utilized lead skull caps. Due to the higher scattered radiation levels at the temple closest to the x-ray gantry [27–30], DoseAware badges were worn as close as practicable to the eye orientated nearer to the x-ray tube. Dosimeters were attached to the skull cap (Fig. 2) or hooked onto the arm of glasses.

A cine and fluoroscopy rate of 15 frames per second (fps) was used for cardiac angiography and implant procedures and 7.5 fps during fluoroscopy in endovascular and electrophysiology cases. Digital subtraction imaging (DSA) post endovascular aneurysm repair (EVAR) deployment was taken at 6 fps. The aortic and pelvic vessels were typically imaged at 3 fps, and rates were incrementally reduced to 0.5 fps for distal leg vessels.

The patient dose parameters of air kerma (AK) (also known as the incident, reference or cumulative air kerma (Ka,r)), and dose area product (DAP) (which may also be referred to as kerma area product (KAP), or air kerma-area product (P_KA_) [31]) were retrieved from procedural dose reports. AK for Philips Allura systems is reported at the international reference point of 15 cm from the isocentre towards the x-ray tube [32]. Direct dosimetry is required to provide accurate reporting of patient dose, but this is impossible to achieve in the clinical setting. It is acknowledged that the use of values provided by indirect dosimetry, such as AK and DAP are subject to uncertainties and may provide a rough estimation only. Reference to ‘patient dose’ is used throughout this manuscript for simplicity.

### Statistical analysis

Eye dose to the operator (cardiologist/vascular surgeon), scrub and circulator nurse, patient AK and DAP were log-normally distributed, and thus they needed to be log-transformed for the analyses. All results of log-transformed variables were exponentiated and reported as geometric means with 95% confidence intervals (CIs). Significant associations were established by non-overlapping 95% CIs. STATA version 15.1 (Stata Corporation, College Station, Texas, USA) and Statistical Discovery Software JMP Pro (Version 15.2.0 SAS Institute, Cary, NC, USA) were used for all analyses.

## Results

**Fig. 3:**
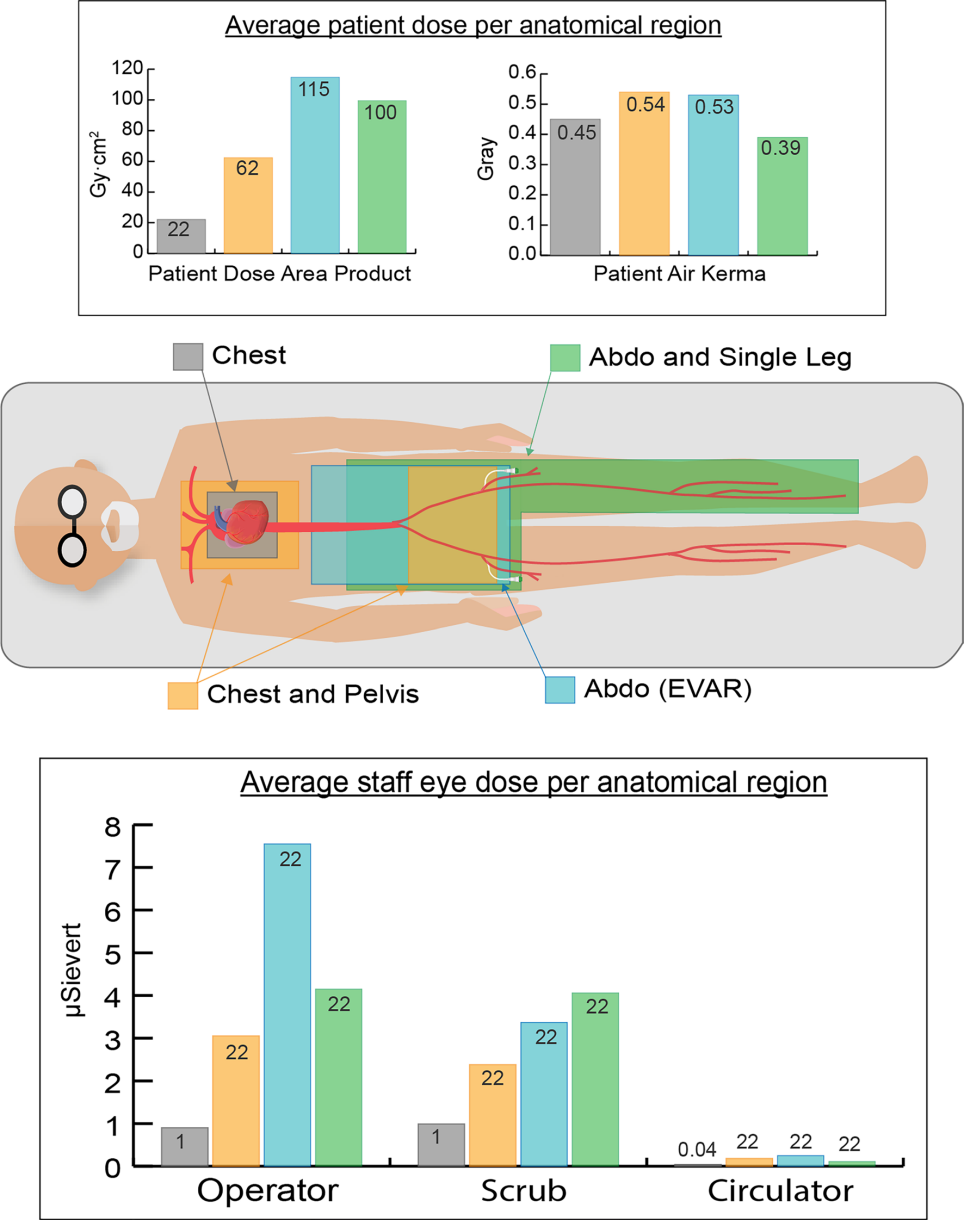
A comparison of average staff eye dose and patient dose during imaging of the anatomical categories of the Chest, Chest + Pelvis, Abdo (non-EVAR) and Abdo and Single Leg. *Abdo* abdomen

**Fig. 4:**
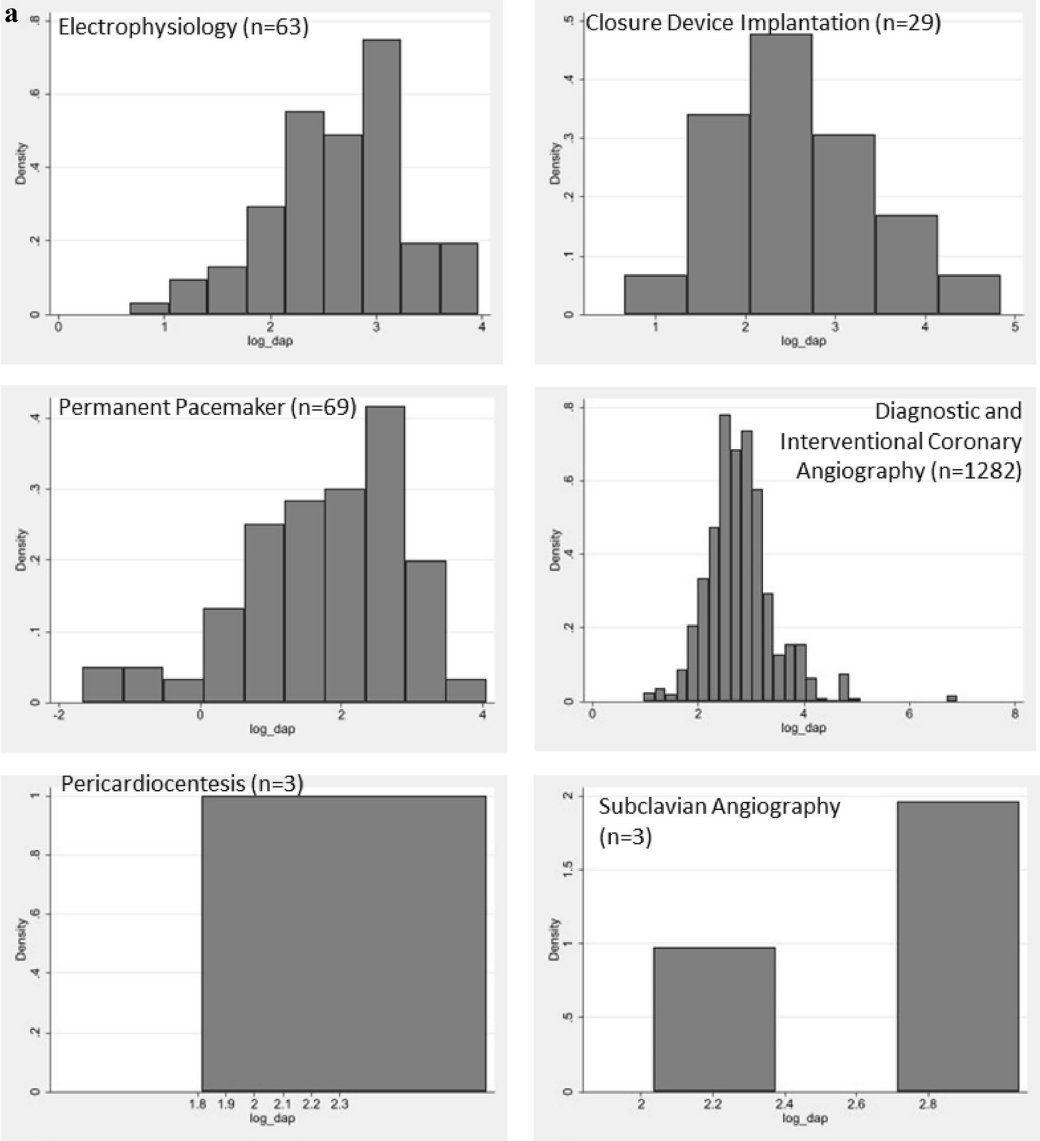

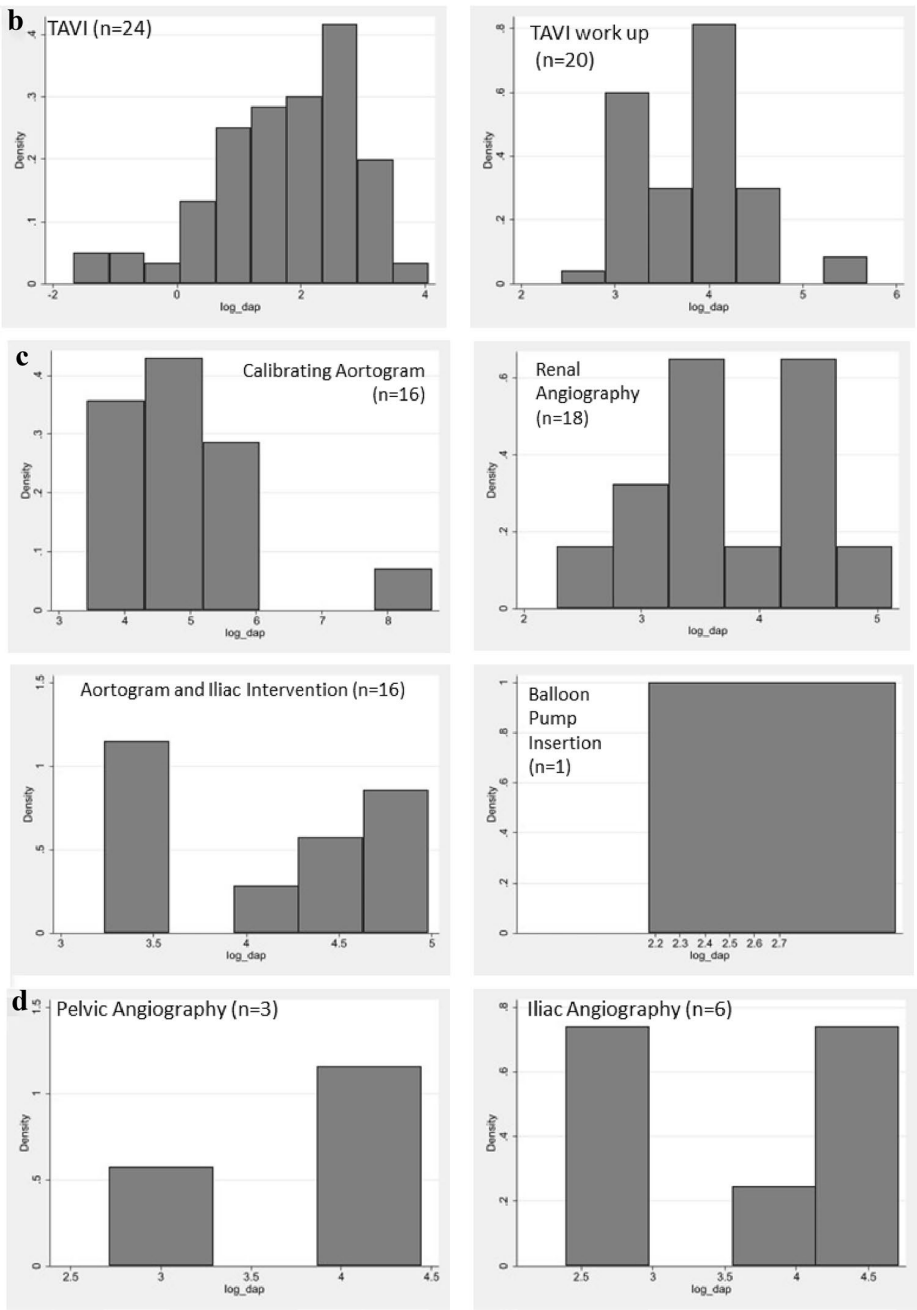
Histograms of DAP during procedural categories included in the anatomical classifications of **a** Chest; **b** Chest + Pelvis; **c** Abdomen (non-EVAR); **d** Pelvis. *TAVI* transcatheter aortic valve implantation

**Fig. 5:**
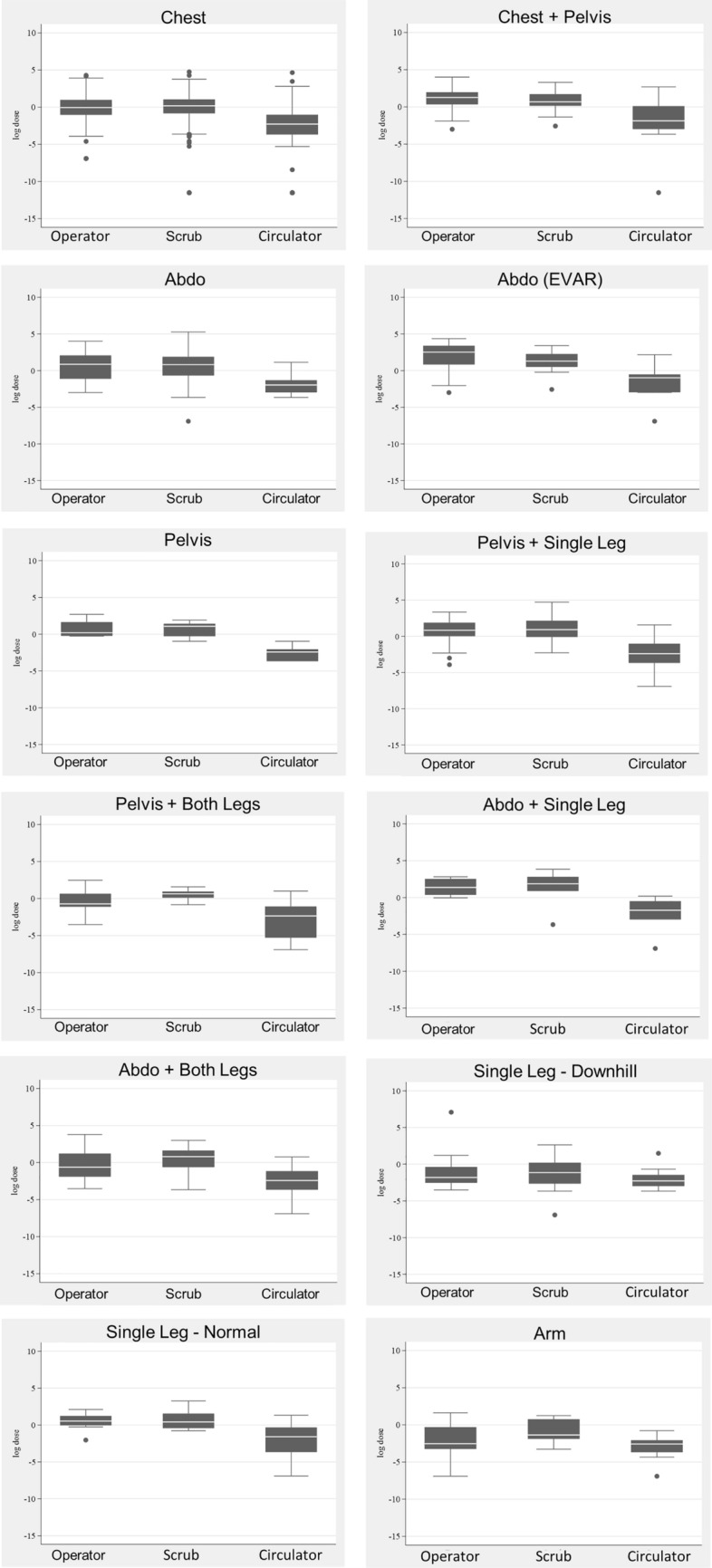
Boxplots of operator, scrub and circulator eye dose. *Dots represent outliers

Dose data were collected for a total of 1792 procedures, with occupational eye dose data readings being available for the operator (n = 1369), scrub nurse (n = 1307) and circulator nurse (n = 885), as demonstrated in Table 2. Figure 3 demonstrates a comparison of occupational and patient dose for the imaging of Chest, Chest + Pelvis, Abdo (non-EVAR) and Abdo + single leg. Due to the range of procedures included in the anatomical categories and potential variations in exposures, Fig. 4 demonstrates histograms of the procedures included in the anatomical categories of Chest, Chest + Pelvis, Abdo (non-EVAR) and Pelvis. The spread of occupational dose is demonstrated in Fig. 5.

**Table 2:** Geometric means (95% CI) of staff eye dose and patient dose measurements for differing types of body area imaged

Procedure	Fluoro time (mins)	Staff dose (μSv)						Patient dose		
		Operator	No of proc N = 1369	Scrub	No of proc N = 1307	Circulator	No of proc N = 885	DAP (Gy•cm^2^)	AK (Gy)	No of proc N = 1792
Chest	5.00 (4.78, 5.24)	0.90 (0.82, 0.98)	1155	0.99 (0.89, 1.09)	1043	0.04 (0.03, 0.06)	706	22.10 (21.21, 23.02)	0.45 (0.43, 0.47)	1455
Chest + pelvis	10.80 (8.30, 14.05)	3.05 (1.53, 6.05)	21	2.38 (1.35, 4.18)	35	0.18 (0.05, 0.64)	27	62.34 (49.29, 78.85)	0.54 (0.42, 0.69)	44
Abdo	3.57 (2.80, 4.56)	1.84 (1.05, 3.24)	31	1.46 (0.88, 2.43)	43	0.17 (0.05, 0.59)	28	70.12 (56.38, 87.22)	0.36 (0.28, 0.45)	51
Abdo (EVAR)	15.03 (11.32, 19.95)	7.55 (4.30, 13.28)	31	3.37 (1.89, 6.02)	33	0.25 (0.06, 0.99)	24	114.80 (89.16, 147.82)	0.53 (0.40, 0.70)	38
Pelvis	4.46 (2.49, 7.97)	2.02 (0.42, 9.71)	4	1.97 (0.65, 6.00)	9	0.08 (0.01, 1.24)	6	41.88 (24.92, 70.41)	0.19 (0.11, 0.32)	9
Pelvis + single leg	5.94 (4.34, 8.13)	1.97 (1.04, 3.74)	24	2.40 (1.25, 4.61)	26	0.09 (0.02, 0.43)	19	30.13 (22.77, 39.85)	0.14 (0.10, 0.18)	31
Pelvis + both legs	2.59 (1.65, 4.06)	0.57 (0.20, 1.63)	9	1.67 (0.51, 5.43)	8	0.05 (0.01, 0.58)	8	23.46 (15.69, 35.08)	0.11 (0.07, 0.18)	15
Abdo + single leg	7.23 (4.27, 12.24)	4.15 (1.37, 12.59)	8	4.06 (1.15, 14.31)	7	0.11 (0.01, 1.36)	7	99.54 (62.22, 159.23)	0.39 (0.23, 0.64)	11
Abdo + both legs	4.13 (3.07, 5.54)	0.86 (0.47, 1.58)	27	1.63 (0.87, 3.07)	28	0.08 (0.02, 0.44)	16	48.26 (37.08, 62.80)	0.21 (0.16, 0.28)	35
Single leg—downhill	3.37 (2.71, 4.19)	0.25 (0.15, 0.43)	36	0.29 (0.18, 0.47)	50	0.13 (0.03, 0.52)	23	5.11 (4.21, 6.21)	0.04 (0.03, 0.05)	64
Single leg—normal	4.64 (3.11, 6.92)	1.68 (0.65, 4.33)	11	1.95 (0.84, 4.48)	16	0.09 (0.01, 0.72)	11	18.41 (12.88, 26.32)	0.08 (0.05, 0.11)	19
Arm	2.46 (1.66, 3.64)	0.12 (0.05, 0.31)	12	0.49 (0.16, 1.48)	9	0.05 (0.01, 0.40)	10	1.81 (1.28, 2.57)	0.02 (0.01, 0.02)	20

*Abdo* abdomen; *AK* air kerma; *DAP* dose area product; *EVAR* endovascular aneurysm repair; *Fluoro* fluoroscopy; *Gy* gray; *Gy•cm*^*2*^ gray centimeter squared; *min* minutes; *μSv* micro sievert

Due to the lengthy duration of such procedures, the average patient DAP was significantly higher during EVAR (114.8 Gy•cm^2^) compared to all other categories, except abdo + SL (99.54 Gy•cm^2^). The average patient AK was also significantly higher during EVAR (0.53 Gy) than during pelvis (0.19 Gy), pelvis + SL (0.14 Gy)/BL (0.11 Gy), abdo + BL (0.21 Gy), SL-DH (0.04 Gy)/N (0.08), or arm (0.02 Gy). As one would expect, imaging of the chest + pelvis resulted in a higher procedural DAP value and was associated with a significantly higher average eye dose to the operator and scrub nurse compared to imaging over the chest only. The mean eye dose to the circulator nurse during chest + pelvis (0.18 μSv) was over four times the dose during chest only imaging (0.04 μSv). The average AK was also higher for the chest and chest + pelvis procedures than all other non-abdo categories.

The average operator eye dose was highest during EVAR (7.55 μSv) and was associated with significantly higher dose when compared with procedures performed in the chest (0.90 μSv), abdo (1.84 μSv), pelvis + SL (1.97 μSv) /BL (0.57 μSv), abdo + BL (0.86 μSv), SL-DH (0.25 μSv), or arm (0.12 μSv). The average eye dose to the operator was the lowest during fistulograms (arm) and SL-DH. Operator eye dose during fistulograms were significantly lower than all categories except pelvis + BL. Similarly, operator eye dose during SL-DH procedures was found to be significantly less than all other procedures excluding those performed on the pelvis and pelvis + BL.

The average eye dose to the scrub nurse was higher than that to the operator during imaging of the chest (0.99/0.90 μSv), pelvis + SL (2.40/1.97 μSv)/BL (1.67/0.57 μSv), abdo + BL (1.63/0.86 μSv), SL-DH (0.29/0.25 μSv) and SL-N (1.95/1.68 μSv). Still, it did not reach statistical significance in any category, as indicated by the overlapping 95% CIs. The average scrub nurse eye dose during EVAR (3.37 μSv) and abdo + SL (4.06 μSv) was significantly increased compared with procedures with imaging of the chest (0.99 μSv), SL-DH (0.29 μSv), or arm (0.49 μSv) (EVAR only).

The average dose to the circulator nurse was lower than both the operator and scrub nurse in all procedural categories. The average eye dose to the circulator nurse was associated with significantly lower dose than the operator and scrub nurse during all procedural categories except Pelvis, Pelvis + BL, SL-DH and arm. The circulator nurse eye dose was also demonstrated to be significantly lower than the operator during abdo + SL, and the scrub nurse during SL-N procedures.

## Discussion

The number and variety of transcatheter vascular procedures performed by numerous medical specialties are increasing, as is the concern regarding the associated radiation risk. There is little current literature comparing the patient dose between the specialties, and there is even less investigating dose to staff other than operators [33–35]. To the best of our knowledge, this is the first clinical study comparing operator, nursing and patient dose for different imaged anatomical locations for fluoroscopically guided cardiac and (non-radiologist) vascular procedures.

Efstathopoulos et al. [36] performed a study which comprised coronary angiography (n = 6), PPM (n = 1), leg angiography (n = 2) and fistula stenting (assumed to be dialysis fistulograms) (n = 2), and is one of the limited comparable studies which included occupational dose to nursing staff. Average procedural operator doses (measured at the left wrist) were higher than the current study (measured at the temple). Recorded doses for Efstathopoulos et al. [36] and the current study, respectively, were 21/1.68 μSv (SL-N) for leg angiography, 33/0.12 μSv for fistulograms, 49/5.1 μSv for pacemakers and 486/0.91 μSv for coronary angiography (isolated data for pacemaker insertion and coronary angiography in the current study not shown). It should be noted that the data collection for the Efstathopoulos et al. [36] study occurred in 2008/2009 using the older style image intensifier imaging equipment and hence would affect the comparison of patient and staff doses utilizing modern equipment and shielding.

Previous authors have investigated differences in patient doses between vascular and radiology specialties. A study by Rigatelli et al. [37] included cardiac and peripheral vascular procedures, but the primary aim was to investigate the effect of operator height rather than comparing doses during differing procedures. Staff dose is also affected by distance from the irradiated area, as found by Sailer et al. and Omar et al., who reported that operator doses during neurology procedures were lower compared to thoracic and abdominal procedures [38, 39]. Bundy et al. reported that interventional neuroradiology had the highest average AK, but that procedures exceeding 5 Gy were more likely performed by vascular surgeons, suggesting this may be due to the lack of radiation training compared to radiologists [40].

This study has demonstrated that the average AK is relatively high for the chest and chest + pelvis procedures. Whilst AK is not an accurate measure of entrance skin dose and does not reflect the effect of tube angulation and collimation, it assists in estimating potential deterministic skin dose effects post-procedure [41]. Coronary angiography, electrophysiology and implantations of closure devices are often performed using magnification to visualize coronary vessels and confirm the positioning of devices. Although increased magnification improves the visualization of smaller anatomical structures, it often comes at the cost of higher patient dose [41], which is reflected in the elevated AK during “chest” imaging in this study. This can be somewhat mitigated by ensuring the detector is as close as safely possible to the patient during screening and acquisitions [41] and utilizing the lowest dose fluoroscopy mode to adequately image anatomy or equipment [42, 43]. Other techniques such as the use of digital magnification have also been demonstrated to reduce patient dose [44].

DAP takes into account the degree of collimation used and reflects the volume of tissue irradiated and hence may be used as an estimator of potential adverse effects [41]. What is of concern is the high average levels of patient DAP, AK, operator and scrub nurse eye dose during EVAR (Fig. 3). Additionally, noting the potential for high occupational doses, care should be taken to minimize the radiation risk posed during EVAR procedures.

Also of interest is the higher average staff and patient dose during chest + pelvis, compared with chest only imaging (Fig. 3). The data for the chest was primarily cardiac procedures (Table 1, Fig. 4), and the procedures in the chest + pelvis category were exclusively composed of TAVIs and TAVI workups. The significantly higher DAP during chest + pelvis may be due to the reduced magnification used during TAVIs and TAVI workups, but it is more likely to result from angiography of the pelvic arteries. It is common at the study center for DSA to be employed for this purpose. It has been previously demonstrated that the use of DSA to image the femoral access point during coronary angiography increases patient dose significantly [45]. Catheter routes are typically imaged via CT pre-TAVI, and consideration should be given to whether there is a genuine need to re-image pelvic arteries using high dose modes such as DSA.

It should also be noted that operator and scrub nurse eye dose is significantly higher during chest + pelvis when compared to procedures which image the chest only. The average circulator nurse dose was also four times higher during chest + pelvis procedures (Table 2). This is potentially due to two causes. The first being a lower average tissue density in the chest due to the presence of the lungs, and the second being the requirement of the circulator nurse to stand close to the patient during the rapid pacing component of TAVI deployment. The circulator nurse utilized an additional moveable lead shield at the study center, but given the dosimeter was worn on the temple, it could be assumed that it did not provide high levels of protection to the head.

**Fig. 6:**
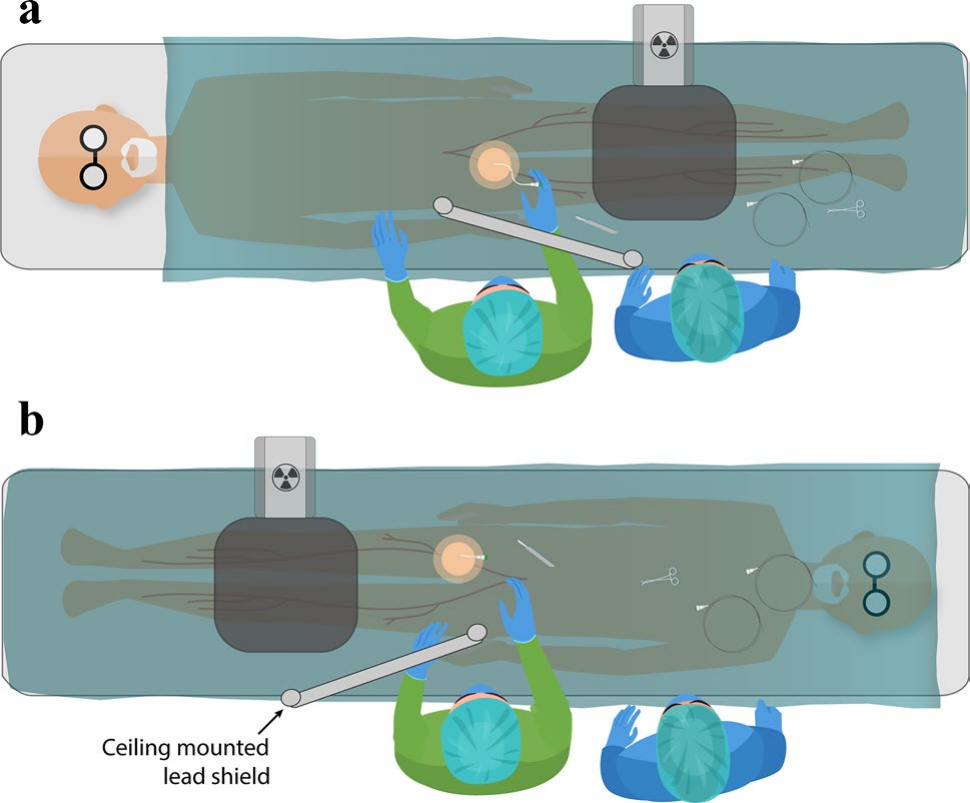
Location of operator and circulator nurse in relation to the x-ray gantry and the ceiling mounted lead shield during; **a** single leg-normal (SL-N); **b** single leg-downhill (SL-DH)

The thickness of the anatomy and the size of the irradiated field alters the levels of scattered radiation [2, 46], as does the average effective atomic number of the scattering tissue [47]. Interestingly, procedures performed on a single leg (SL-N) resulted in a similar average operator head dose as those imaging the abdo, pelvis, pelvis + SL, and was surprisingly higher than the chest, pelvis + BL and abdo + BL. This is most likely due to the proximity of the staff to the imaged area, which has been demonstrated previously to affect operator dose [39]. This is evidenced further by the eye dose to both the operator and scrub nurse being related to significantly lower dose during the downhill approach, which allows for increased distance from the x-ray tube, as demonstrated in Fig. 6. The positioning of the ceiling-mounted lead shield has also been shown to influence dose levels to the nursing staff, potentially leading to higher dose to the scrub nurse compared to the operator [48]. This may explain the average temple dose to scrub nurses being four times that of the operator during imaging of the arm.

The majority of previous studies have indicated that occupational doses to operators are significantly higher than to other staff [30, 34, 35]. The results in this study indicated that the average eye dose to the operator and to the scrub nurse was significantly higher than to the circulator nurse during procedures of the chest, chest + pelvis, abdo, EVARs, pelvis + SL, and abdo + SL (operator only), abdo + BL. The differences between the operator and scrub nurse dose did not reach significance, but the spread of doses should be highlighted (Fig. 5). In fact, the average dose to the scrub nurse was higher than to the operator during procedures of the chest, pelvis + SL/BL, abdo + BL, arm and SL-DH/N. It is worthy of noting that of the 12 procedural categories included in this study, the average eye dose to the scrub nurse was higher than the operator during 7, and similar for the remaining 5.

The patient and occupational ‘eye’ doses measured within this study are lower than reported in contemporary investigations of fluoroscopically guided cardiac and vascular procedures [49–53]. It should be noted that the dosimetry badges were worn external to the protective apparel and hence do not reflect the actual levels of radiation incident on the tissues of the eye. Additionally, when doses were extrapolated to estimate yearly doses, they fell well below the current recommended eye dose limits of 20 mSv/year [54].

The single-center design of this study, additional lead shielding and the specific procedural protocols employed may limit the generalizability of the results. However, this could also be seen as a strength as the same nursing and medical imaging staff performed both the cardiology and vascular procedures, and the same equipment was also utilized for the study. This has the advantage of limiting the variables present in similar studies when comparing procedures performed by differing medical specialities. An additional limitation is that patient and staff dose is also affected by numerous imaging factors such as collimation and magnification. It is difficult to collect data on these parameters due to their variations within procedures in the clinical setting and was beyond the scope of this study. Still, as noted previously, the same staff were involved in procedures, lending a degree of consistency across procedures in terms of the use of collimation and magnification. A further limitation is that the correlation between anatomical areas may have been influenced by the significantly higher number of cases in the chest category. Additionally, the manufacturer specifications state that DoseAware detects scatter radiation down to 1 μSv. This study has reported doses < 1 μSv which may lead to greater levels of uncertainty in these measurements.

## Conclusion

This research compared patient and staff dose levels during fluoroscopic procedures performed within the chest, abdo, pelvis, arms and legs by cardiologists and vascular surgeons. EVAR were found to contribute the highest average dose to both staff and patients. The use of DSA to image the pelvic arteries during TAVIs and TAVI workups increased patient DAP as well as dose to the operator and scrub nurse, hence DSA acquisitions should be minimised to keep radiation dose to staff and patients as low as reasonably achievable.. Personnel performing rapid pacing during TAVIs should be aware of a potential increase in dose to the eye. In addition, staff should be aware that the eye dose to the scrub nurse has the potential to exceed the operators.

## Data Availability

All data referred to and underpinning this publication are openly available in QUT Research Data Finder and can be found at https://researchdatafinder.qut.edu.au/display/n11970.
